# Association of the Onset of Self-Feeding With Subsequent Suspected Developmental Coordination Disorder: A Prospective Cohort Study in China

**DOI:** 10.3389/fpsyt.2022.818771

**Published:** 2022-05-06

**Authors:** Jing Hua, Gareth J. Williams, Anna L. Barnett, Jiajia Zhang, Hua Jin, Manyun Xu, Juan Chen, Yingchun Zhou, Guixiong Gu, Wenchong Du

**Affiliations:** ^1^Shanghai Key Laboratory of Maternal Fetal Medicine, Shanghai First Maternity and Infant Hospital, School of Medicine, Tongji University, Shanghai, China; ^2^School of Social Sciences, Nottingham Trent University, Nottingham, United Kingdom; ^3^Centre for Psychological Research, Oxford Brookes University, Oxford, United Kingdom; ^4^Health Care Department of Suzhou Ninth People’s Hospital, Suzhou, China; ^5^KLATASDS-MOE, School of Statistics, East China Normal University, Shanghai, China; ^6^Pediatrics Research Institution of Suzhou University, Suzhou, China; ^7^NTU Psychology, Nottingham Trent University, Nottingham, United Kingdom

**Keywords:** self-feeding, early behavioral marker, developmental coordination disorder, preschool children, two-child families

## Abstract

**Background:**

Successful self-feeding reflects the readiness of early motor development and environmental impacts, and the onset of self-feeding as a developmental milestone might be a predictor of subsequent motor development in children. In this study, we explored the association between the onset of self-feeding and childhood risk of Developmental Coordination Disorder in children from one-child and two-child families.

**Methods:**

We conducted a data-linkage prospective cohort study from 38 kindergartens in 6 cities in China. A total of 11,727 preschoolers aged 3–6 years old were included in the final analysis and were assessed with the Movement Assessment Battery for Children-second edition (MABC-2) Test. The information on early self-feeding onset was obtained from parents. The mixed and multi-level logistic models utilizing a random intercept were used to investigate the associations between the onset time of self-feeding and subsequent motor performance.

**Results:**

The results showed that, compared with those beginning self-feeding at or younger than 12 months of age, children starting self-feeding at 13–24, 25–36, and later than 36 months, showed a decrease in their total MABC-2 scores of 2.181, 3.026, and 3.874, respectively; and had an increased risk of suspected DCD by 36.0, 101.6, 102.6%, respectively; they also had 30.2, 46.6, 71.2% increased prevalence of at risk of suspected DCD, when adjusting for both child and family characteristics (each *p* < 0.05). Significant associations were observed in fine motor, gross motor, and balance subtests (each *p* < 0.05) in groups with a delayed onset of self-feeding. However, the strength of the associations was mitigated in the fine motor and balance subtests in children with a sibling.

**Conclusion:**

The delayed onset time of self-feeding acts as an early behavioral marker for later childhood motor impairment. Moreover, children with a sibling may benefit from additional interaction and their motor developmental pattern may be affected by the presence of a sibling.

## Introduction

The onset time of attainment of motor milestones in infancy can be influenced by both physiological and environmental factors (1). The World Health Organization (WHO) developed normal age ranges for the achievement of motor milestones among healthy children (2), which parents and healthcare professionals can use as benchmarks to flag children at risk of developmental delays or help identify late achievers. Children with Developmental Coordination Disorder (DCD) experience marked motor impairment, and one of the diagnostic criteria of DCD emphasizes that the symptoms of DCD should start to reveal from early childhood (3), which means the symptoms of DCD are usually apparent in their early years.

The individual motor profile of each child with DCD may vary widely, but the disorder manifests itself in disruption to a wide range of everyday activities (4, 5), which can negatively affect children’s daily activities such as eating, dressing, and personal hygiene (6, 7). Children with DCD are often observed to display poor eating skills (7), and tend to be described as messy eaters (6, 8), given their impairment in motor and coordination skills required in using cutlery in eating (6, 9). When assessed with food preparation tasks, such as putting on an apron, making a sandwich, and preparing chocolate milk (requiring children to prepare and mix milk with chocolate powder), children with DCD also showed significantly lower performance scores compared with typically developing children (8). Moreover, observed and self-reported difficulties were also reported in children with DCD in their self-feeding activities (6, 10).

Self-feeding, defined as using cutlery to eat independently, is a complex activity that engages various movements, including arm and finger movements, chewing and swallowing, and using cutlery (11). Self-feeding is therefore a developmental milestone in early childhood that reflects the developmental readiness of fine- and gross-motor skills, along with hand-eye coordination (12, 13), which can be easily noticed and recorded by parents and childcare professionals. An association between the onset time of self-feeding and later motor development can not only provide direct evidence to understand how the motor milestone in early childhood predicts later development of different domains of motor skills, but can also help us better understand to what extent the delays in early motor activities can facilitate the early identification of DCD.

Self-feeding skills can also be influenced significantly by environmental factors such as parental styles (14–16) and the interaction with a sibling (14, 17). The presence of a sibling can introduce social learning and peer competition (18), which can encourage self-feeding behaviors and positively influence the development of self-feeding skills. The association between the early self-feeding achievement and later motor impairment might be different between children with and without siblings. China relaxed its birth-control policy in 2016 allowing all families to have two children. As a consequence, there are increasing numbers of families having more than one child in China. However, the parents themselves were from the one-child generation and may not have much experience in observing or interacting with a sibling, which can affect their parenting style (19) and affect their children’s development as a consequence (20). As such, the role of the presence of a sibling in the association between self-feeding onset time and suspected DCD was also examined in one-child and multi-child families.

The current study, therefore, aimed to investigate the association between the onset of self-feeding and subsequent suspected DCD in early childhood. We hypothesized that the onset of self-feeding is associated with children’s later fine- and gross- motor performance, and hand-eye coordination. Confounding factors, including child and family characteristics that can influence motor development, were controlled (21–24). The role of the presence of a sibling in the association between self-feeding onset and motor development was also examined.

## Materials and Methods

### Population

We conducted a population-based cohort study with data from six cities in Jiangsu Province in southeast China. The data were derived from the healthcare database of the local health care institutions, where the data of each child’s physical and neurobehavioral outcomes during infancy and toddlerhood was collected between March 1st 2010 and January 31st 2012. The children in the database were then followed up with assessments in their preschool period. Children with physical disabilities, intellectual impairment, or any severe developmental disorder (e.g., autism) according to their clinical records were excluded from the study.

**FIGURE 1:**
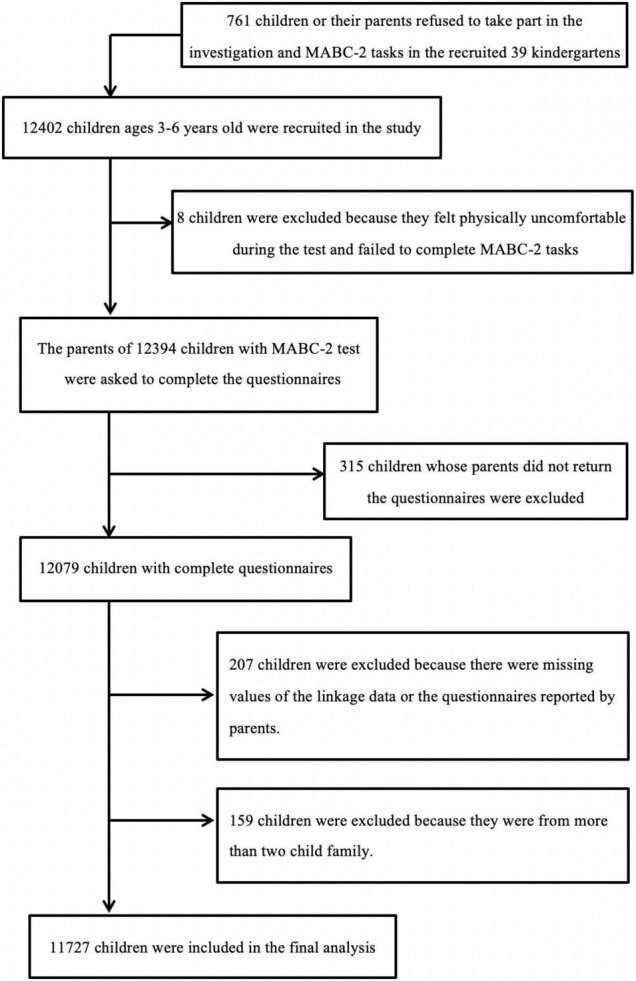
Flowchart of the study population.

A total of 12,402 children aged 3–6 years old from 38 kindergartens were included in the study. Some children were removed from the analysis due to failing to complete the tests or having missing values. The children from families with more than two children were also excluded from the study^1^. A total of 11,727 children were included in the final analysis (Figure 1).

The study was approved by the local Education Board and Ethics Committee of the Children’s Hospital of Suzhou University. Parental consent and children’s assent was obtained before the investigation and tests. All information acquired was kept confidential and was accessible only to the researchers.

### Outcomes, Predictors, and Covariates

The Movement Assessment Battery for Children-2nd edition (MABC-2) Test is a globally recognized assessment to identify children with motor impairments (25). MABC-2 has been reported to have high validity and reliability in Chinese children (26). Age band 1 of the MABC-2 test for children aged 3–6 years old was used in the study. The test contains eight tasks categorized into the following three motor domains: 3 tasks measure Manual Dexterity skills (MD) (posting coins; threading lace; drawing); 2 tasks measure Aiming and Catching skills (AC) (throwing/aiming and catching a beanbag); and 3 tasks measure Balance skills (BAL) (two-leg balance; walking along a line; jumping). For each task, a raw score was obtained and was then converted to a standard score according to the original test manual. Higher test scores indicate better motor performance. Children’s motor performance was indicated by the total scores of MABC-2 and scores on each of the three components (Manual Dexterity, Aiming and Catching, and Balance). According to the DSM-V, DCD should be diagnosed based on the following criteria:

(i) acquisition and execution of coordinated motor skills are below the expected level for age, given the opportunity for skill learning; (ii) motor skill difficulties significantly interfere with activities of daily living and impact academic/school productivity, prevocational and vocational activities, leisure, and play; (iii) onset is in the early developmental period; and (iv) motor skill difficulties are not better explained by intellectual or visual impairment or other neurological conditions that affect movement. MABC-2 was therefore used in the current study to measure the significant motor impairment (criteria i) which is considered as the most important criteria to define DCD. Children who obtained a total standard score on the MABC-2 Test below the 5th percentile were considered to have significant movement difficulties and below the 15th percentile were considered to have mild movement difficulties, in line with DCD. Children achieving a score below the 6th and 15th percentile on the MABC-2 Test were therefore identified as children “with suspected DCD” (<6%), and “at risk of suspected DCD” (6–16%), because the other three criteria as described earlier were not directly tested using MABC-2. The children achieving a score higher than 16% were defined as children with “typical performance” according to the MABC-2 Examiner’s Manual (27).

The information on children’s eating behavior and other potential confounders (gender, handedness, eyesight, gestational weeks, birth weight, weight, and height) was collected from the database of the local health care institutions. Self-feeding was defined as the age in months when a child was able to eat by themselves with any cutlery (including either a spoon, fork, or chopsticks), and the information was provided by the parents.

We included a range of child, family, and maternal health characteristics as potential confounders according to the literature (Table 1): (1). Child characteristics included the child’s age, sex, child body mass index (BMI), right-handedness, eyesight, gestational weeks, birth weight. BMI is an indicator of obesity that is based on height and weight [BMI = weight(kg)/height(m)]. The children’s height was measured by a metric stadiometer attached to the wall; their weight was measured using a digital scale. Eyesight was grouped into normal and abnormal (including myopia, hyperopia, astigmatism, etc.) (2). Family characteristics were collected from a parent self-report questionnaire, which included the education level of the mother and father, the family’s annual per capita income, the number of children in the family, maternal age of the mother, maternal complications during pregnancy, and family structure. Family structure was classified into three types, “three-generation (or more) family,” “nuclear family (both parents and children),” and “single-parent family.” The “Three-generation (or more) family” refers to a child living with their parents and grandparents, which is a common family structure in Chinese communities (3). Maternal characteristics included the following variables: maternal age at delivery (<30, 30–34, and ≥35 years); Maternal complications were defined according to the International Classification of Diseases, Revision 10 (ICD 10). The classification is defined as having one of the following maternal complications during pregnancy: vaginal bleeding during pregnancy, risk of miscarriage, use of antibiotics, use of fertility drugs, intrauterine distress, and fetal asphyxia.

**TABLE 1:** Total and component MABC-2 scores by child and family characteristics (Mean ± SD)*^a^*.

Characteristic	Total score	Manual dexterity	Aiming and catching	Balance
**Child characteristics**				
**Child age (years)**				
3	103.486 ± 13.741***	29.013 ± 4.810**	17.774 ± 5.996***	35.927 ± 5.383***
4	101.055 ± 13.631	27.599 ± 5.193	17.961 ± 5.344	35.271 ± 4.858
5	107.663 ± 12.885	30.174 ± 6.644	19.875 ± 4.518	35.393 ± 3.501
6	103.102 ± 12.447	28.760 ± 5.615	19.162 ± 5.041	33.797 ± 3.141
**Gender**				
Male	103.135 ± 13.878***	28457 ± 6.078***	19.051 ± 5.142**	34.585 ± 4.368***
Female	105.208 ± 12.664	29.471 ± 5.588	18.703 ± 5.154	35.530 ± 3.860
**BMI of the child**				
≤18	104.061 ± 13.376	28.898 ± 5.871	18.855 ± 5.158**	35.043 ± 4.172*
>18	104.197 ± 13.493	29.156 ± 6.008	19.320 ± 5.050	34.632 ± 4.172
**Right handedness**				
No	104.092 ± 13.353	28.937 ± 5.868	18.897 ± 5.141	35.012 ± 4.165
Yes	103.686 ± 14.029	28.437 ± 6.203	18.795 ± 5.380	35.047 ± 4.337
**Eyesight**				
Normal	104.302 ± 13.280***	28.961 ± 5.8478**	18.933 ± 5.148***	35.085 ± 4.121***
Abnormal	101.924 ± 14.127	28.499 ± 6.199	18.515 ± 5.155	34.336 ± 4.573
**Gestational weeks**				
<37	103.153 ± 13.081	28.979 ± 5.968	18.286 ± 4.775**	34.660 ± 4.008*
≥37	104.150 ± 13.402	28.912 ± 5.876	18.942 ± 5.176	35.042 ± 4.183
**Birth weight**				
<2500 g	105.202 ± 13.085	29.735 ± 6.083	19.116 ± 4.992	35.006 ± 3.846
≥2500 g	104.041 ± 13.388	28.892 ± 5.875	18.886 ± 5.155	35.014 ± 4.181
Family characteristics				
**Higher education of the mother**				
No	103.648 ± 13.379***	28.510 ± 6.045***	18.677 ± 5.097*	35.100 ± 4.117***
Yes	104.443 ± 13.372	29.267 ± 5.717	19.078 ± 5.188	34.940 ± 4.217
**Higher education of the father**				
No	103.638 ± 13.604*	28.500 ± 6.176***	18.632 ± 5.086	35.099 ± 4.147***
Yes	104.349 ± 13.232	29.178 ± 5.676	19.056 ± 5.184	34.960 ± 4.186
**Family annual per-capita income (RMB)*^b^***				
Below	103.541 ± 13.341***	28.840 ± 5.965***	18.475 ± 4.898***	34.879 ± 4.231***
Above or equal to	105.446 ± 13.384	29.114 ± 5.662	19.963 ± 5.605	35.359 ± 3.995
**Family structure**				
Single families	102.271 ± 14.226	28.581 ± 7.017	18.465 ± 4.470	34.369 ± 4.630**
Nuclear families	104.000 ± 13.419	28.795 ± 5.926	18.867 ± 5.158	35.059 ± 4.115
Extended families	104.282 ± 13.277	29.160 ± 5.741	18.953 ± 5.162	34.951 ± 4.256
**Number of children in the family**				
One	104.190 ± 13.426	29.002 ± 5.879	18.974 ± 5.153	34.974 ± 4.191***
Two	103.617 ± 13.187	28.575 ± 5.885	18.566 ± 5.128	35.172 ± 4.091
**Maternal age at birth**				
<30	104.113 ± 13.350	28.906 ± 5.864	18.915 ± 5.164*	35.029 ± 4.175
30–34	104.288 ± 13.414	29.111 ± 5.971	18.895 ± 5.042	35.000 ± 4.068
≥35	102.402 ± 13.937	28.516 ± 6.026	18.322 ± 5.152	34.680 ± 4.423
**Maternal complications during pregnancy*^c^***				
No	104.223 ± 13.493*	28.932 ± 5.968*	18.954 ± 5.155	35.041 ± 4.143
Yes	103.455 ± 12.877	28.851 ± 5.507	18.633 ± 5.122	34.897 ± 4.289

*^a^One-way ANOVA.*

*^b^The national average family per-capita income in the last year of the survey time.*

*^c^Having one of the maternal complications during pregnancy including vaginal bleeding during pregnancy, threatened miscarriage, use of antibiotics, use of fertility drugs, intrauterine distress, fetal asphyxia.*

*p < 0.05, **p < 0.01, ***p < 0.00.

### Data Analysis

Chi-square analyses were used to compare children’s age, gender, BMI, parents’ education and family income between the children with motor impairment, at-risk of motor impairment and those with typical motor performance. One-way ANOVAs were used to compare the mean MABC-2 scores by child and family characteristics.

According to previous studies, the relationship between early motor development and later neuro-behavioral performance is linear in most cases (28, 29). The mixed model utilizing a random intercept (we considered kindergarten as a cluster and hypothesized that there was no interaction between kindergartens and self-feeding) was used to investigate the associations between timing of self-feeding and MABC-2 scores, when adjusting for the clustering (kindergartens) and other potential confounders (mother and child characteristics). Adjusted odds ratios were estimated to determine the strength of association for the home and educational environment with poor motor performance (0 = typical performance, 1 = at-risk of suspected DCD, 2 = suspected DCD) using a multilevel logistic regression model. All analyses were performed in R 2.15. using the ‘MGCV’ and ‘LME4.’ A *p* < 0.05 was considered statistically significant. Furthermore, when measuring the association between the timing of self-feeding and motor impairment, we carried out a sensitivity analysis to compare the difference of the association when including all participants and excluding the children whose families had more than two children.

## Results

Of 11,727 children, 4,694 (40.0%) began to self-feed at less than 12 months of age. For the rest, 4,744 (40.4%), 2,035 (17.4%), and 254 (2.2%) started to feed independently in age bands of 13–24, 25–36, and >36 months, respectively. The MABC-2 test scores by child and family characteristics are shown in Table 1. The rates of suspected DCD, at-risk of suspected DCD and typical performance by child and family characteristics, are shown in Table 2 and Supplementary Table 1.

**TABLE 2:** Rates of motor impairment by child and family characteristics (*n* = 11727)*^a^*.

Characteristic	Suspected DCD (≤5th centile of MABC-2)	At-risk of suspected DCD (6–16th centiles of MABC-2)	Typical performance >16th centile of MABC-2
**Child characteristics**			
**Child age (n %)**			
3	47 (9.50)***	142 (12.1)	873 (8.7)
4	232 (47.1)	471 (40.0)	2763 (27.5)
5	95 (19.3)	263 (22.4)	3986 (39.6)
6	119 (24.1)	300 (25.5)	2436 (24.2)
**Gender (n %)**			
Male	336 (68.2)***	727 (61.9)	5396 (53.6)
Female	157 (31.8)	448 (38.1)	4663 (46.40)
**Present BMI (n %)**			
≤18	454 (92.3)	1095 (93.1)	9098 (90.4)
>18	38 (7.7)	81 (6.9)	961 (9.6)
**Right handedness (n %)**			
No	472 (95.7)	1125 (95.7)	9625 (95.7)
Yes	21 (4.3)	50 (4.3)	434 (4.3)
**Eye-sight (n %)**			
Normal	425 (86.2)***	1037 (88.3)	9018 (89.7)
Abnormal	68 (13.8)	138 (11.7)	1041 (10.3)
**Gestational weeks (n %)**			
<37	43 (8.7)	93 (7.9)	813 (8.1)
≥37	450 (91.3)	1082 (92.1)	9246 (91.9)
**Birth weight (n %)**			
<2500 g	10 (2.0)	33 (2.8)	463 (4.6)
≥2500 g	483 (98.0)	1143 (97.2)	9595 (95.4)
**Family characteristics (n %)**			
**Higher education of mother**			
No	255 (51.7)***	540 (46.0)	4657 (46.3)
Yes	238 (48.3)	635 (54.0)	5402 (53.7)
**Higher education of father (n %)**			
No	231 (46.9)*	449 (38.2)	3716 (36.9)
Yes	262 (53.1)	726 (61.8)	6343 (63.1)
**Family annual per-capita income (n %) (RMB)*^b^***			
Below	387 (78.5)***	880 (74.9)	7307 (72.6)
Above or equal to	106 (21.5)	295 (25.1)	2752 (27.4)
**Family structure (n %)**			
Single families	6 (1.2)	24 (2.0)	127 (1.3)
Nuclear families	328 (66.5)	759 (64.6)	6421 (63.8)
Extended families	159 (32.3)	392 (33.4)	3511 (34.9)
**Number of children in the family (n %)**			
One	389 (78.9)**	950 (80.9)	8111 (80.6)
Two	104 (21.1)	225 (19.1)	1948 (19.4)
**Maternal age at birth (n %)**			
<30	416 (84.4)	1010 (85.9)	8621 (85.7)
30–34	52 (10.5)	127 (10.8)	1156 (11.5)
≥35	25 (5.1)	39 (3.3)	281 (2.8)
**Maternal complications during pregnancy*^c^* (n %)**			
No	394 (79.9)	954 (81.2)	8280 (82.3)
Yes	99 (20.1)	221 (18.8)	1779 (17.7)

*^a^Pearson chi-square.*

*^b^The national average family per-capita income in the last year of the survey time.*

*^c^Having one of maternal complications during pregnancy including vaginal bleeding during pregnancy, threatened miscarriage, use of antibiotics, use of fertility drugs, intrauterine distress, fetal asphyxia.*

*p < 0.05, **p < 0.01, ***p < 0.001.

As shown in Table 3, when compared with those beginning self-feeding at or younger than 12 months of age, children starting self-feeding at 13–24, 25–36, and more than 36 months, there was a decrease in total MABC-2 scores of 2.181, 3.026, and 3.874, respectively, when adjusting for both child and family characteristics (each *p* < 0.05). Children who began to eat independently at 13–24, 25–36, and more than 36 months of age were associated with MABC-2 scores of manual dexterity (β = −0.873, −1.012, and −1.492, respectively, each *p* < 0.05) and aiming and catching (β = −1.109, −1.558, and −1.497, respectively, each *p* < 0.05), when adjusting for both child and family characteristics (each *p* < 0.05). Children who began to eat independently at 25–36 and more than 36 months were associated with a drop in balance scores (β = −0.565 and −0.676, respectively, each *p* < 0.05) when adjusting for both child and family characteristics (Table 3). The association between 1 month delay of self-feeding and MABC-2 scores were shown in Table 3.

**TABLE 3:** Associations between the onset of self-feeding and motor performance in pre-schoolers (*n* = 11727).

	Crude β*^a^* (95% CI)	Adjusted β*^b^* (95% CI)	Adjusted β*^c^* (95% CI)	Adjusted β*^d^* (95% CI)
**MABC-2 Total score**				
Month age	−0.138 (−0.176∼−0.100)***	−0.127 (−0.165∼−0.089)***	−0.126 (−0.166∼−0.086)***	−0.114 (−0.154∼−0.074)***
≤12 month age	Ref	Ref	Ref	Ref
13–24 month age	−2.482 (−3.348∼−1.616)***	−2.445 (−3.301∼−1.588)***	−2.256 (−3.220∼−1.291)***	−2.181 (−3.149∼−1.214)***
25–36 month age	−3.559 (−4.672∼−2.446)***	−3.302 (−4.422∼−2.183)***	−3.309 (−4.540∼−2.079)***	−3.026 (−4.273∼−1.779)***
>36 month age	−4.455 (−6.430∼−2.479)***	−4.147 (−6.103∼−2.190)***	−4.194 (−6.172∼−2.216)***	−3.874 (−5.834∼−1.913)***
**Manual dexterity**				
Month age	−0.041 (−0.054∼−0.028)***	−0.037 (−0.050∼−0.024)***	−0.043 (−0.057∼−0.029)***	−0.038 (−0.053∼−0.024)***
≤12 month age	Ref	Ref	Ref	Ref
13–24 month age	−0.809 (−1.161∼−0.457)***	−0.801 (−1.159∼−0.444)***	−0.896 (−1.316∼−0.477)***	−0.873 (−1.296∼−0.449)***
25–36 month age	−1.020 (−1.405∼−0.635)***	−0.930 (−1.325∼−0.535)***	−1.117 (−1.551∼−0.683)***	−1.012 (−1.456∼−0.567)***
>36 month age	−1.543 (−2.351∼−0.736)***	−1.425 (−2.232∼−0.619)***	−1.619 (−2.444∼−0.793)***	−1.492 (−2.317∼−0.668)***
**Aiming and catching**				
Month age	−0.069 (−0.086∼−0.053)***	−0.069 (−0.086∼−0.053)***	−0.057 (−0.073∼−0.040)***	−0.056 (−0.072∼−0.039)***
≤12 month age	Ref	Ref	Ref	Ref
13–24 month age	−1.409 (−1.731∼−1.087)***	−1.440 (−1.760∼−1.120)***	−1.119 (−1.456∼−0.783)***	−1.109 (−1.453∼−0.765)***
25–36 month age	−1.899 (−2.369∼−1.429)***	−1.893 (−2.360∼−1.426)***	−1.606 (−2.075∼−1.137)***	−1.558 (−2.033∼−1.083)***
>36 month age	−1.778 (−2.505∼−1.051)***	−1.839 (−2.554∼−1.123)***	−1.474 (−2.190∼−0.759)***	−1.497 (−2.208∼−0.787)***
**Balance**				
Month age	−0.030 (−0.044∼−0.017)***	−0.027 (−0.040∼−0.014)***	−0.025 (−0.038∼−0.011)***	−0.023 (−0.036∼−0.010)***
≤12 month age	Ref	Ref	Ref	Ref
13–24 month age	−0.416 (−0.703∼−0.129)**	−0.360 (−0.645∼−0.075)*	−0.299 (−0.594∼−0.004)*	−0.284 (−0.575∼0.008)
25–36 month age	−0.743 (−1.082∼−0.405)***	−0.663 (−1.000∼−0.325)***	−0.597 (−0.953∼−0.242)***	−0.565 (−0.920∼−0.210)**
>36 month age	−0.930 (−1.624∼−0.236)**	−0.748 (−1.412∼−0.083)*	−0.807 (−1.489∼−0.125)*	−0.676 (−1.332∼−0.019)*

*^a^Not adjusted for other variables.*

*^b^Adjusted for child characteristics.*

*^c^Adjusted for family characteristics.*

*^d^Adjusted for child and family characteristic.*

*p < 0.05, **p < 0.01, ***p < 0.001.

As shown in Table 4, children started self-feeding at 13–24, 25–36 and, more than 36 months had an increased risk of overall motor impairment – as measured by the total MABC-2 scores – of 36.0, 101.6, 102.6% for the suspected DCD group, and 30.2, 46.6, 71.2% for the at-risk of suspected DCD group when compared with typically performing children when adjusting for both child and family characteristics (each *p* < 0.05). Children who began eating independently at 13–24, 25–36, and more than 36 months of age were associated with increased risks of significant fine motor impairment (adjusted OR = 1.452, 1.855, and 2.000, respectively), gross motor impairment (adjusted OR = 1.315, 1.788, and 2.051, respectively) for the suspected DCD group when compared with typically performing children (each *p* < 0.05). Children who had a self-feeding onset of 13–24, 25–36 and more than 36 months of age were associated with an increased risk of fine motor impairment (adjusted OR = 1.255, 1.286, and 1.271, respectively) and gross motor impairment (adjusted OR = 1.368, 1.468, and 1.601, respectively) for the at-risk of suspected DCD group when compared to typically performing children (each *p* < 0.05). However, there was no observed statistically significant risk of balance impairment in almost all age bands, when adjusted for child and family characteristics (Table 4). The association between 1 month delay of self-feeding and the risk of DCD were shown in Table 4.

**TABLE 4:** Associations between the onset of self-feeding and suspected DCD in pre-schoolers (*n* = 11727).

Characteristic	Suspected DCD vs. Typical performance				At-risk of suspected DCD vs. Typical performance			
	cOR*^a^* (95% CI)	aOR*^b^* (95% CI)	aOR*^c^* (95% CI)	aOR*^d^* (95% CI)	cOR*^a^* (95% CI)	aOR*^b^* (95% CI)	aOR*^c^* (95% CI)	aOR*^d^* (95% CI)
**Overall motor impairment**								
Month age	1.026 (1.016∼1.036)***	1.024 (1.014∼1.034)***	1.025 (1.014∼1.036)***	1.023 (1.012∼1.033)***	1.017 (1.010∼1.024)***	1.016 (1.008∼1.023)***	1.016 (1.009∼1.024)***	1.015 (1.007∼1.022)***
≤12 month age	Ref	Ref	Ref	Ref	Ref	Ref	Ref	Ref
13–24 month age	1.415 (1.081∼1.851)*	1.409 (1.077∼0.1842)*	1.377 (1.033∼1.834)*	1.360 (1.021∼1.812)*	1.317 (1.099∼1.579)**	1.313 (1.097∼1.572)**	1.315 (1.074∼1.610)**	1.302 (1.064∼1.594)*
25–36 month age	2.184 (1.666∼2.862)***	2.086 (1.589∼2.738)***	2.122 (1.587∼2.836)***	2.016 (1.506∼2.700)***	1.543 (1.254∼1.899)***	1.497 (1.216∼1.844)***	1.522 (1.212∼1.910)***	1.466 (1.166∼1.843)***
>36 month age	2.189 (1.175∼4.078)*	2.069 (1.118∼3.830)*	2.153 (1.140∼4.068)*	2.026 (1.080∼3.801)*	1.761 (1.168∼2.656)**	1.709 (1.133∼2.580)*	1.770 (1.179∼2.657)**	1.712 (1.139∼2.572)**
**Fine motor impairment** (**Manual dexterity**)								
Month age	1.022 (1.012∼1.032)***	1.021 (1.011∼1.030)***	1.023 (1.014∼1.033)***	1.022 (1.013∼1.032)***	1.009 (1.002∼1.015)*	1.007 (1.000∼1.014)*	1.009 (1.002∼1.017)*	1.008 (1.000∼1.015)*
≤12 month age	Ref	Ref	Ref	Ref	Ref	Ref	Ref	Ref
13–24 month age	1.358 (1.024∼1.800)*	1.359 (1.030∼1.793)*	1.443 (1.066∼1.954)*	1.452 (1.071∼1.970)*	1.236 (1.038∼1.471)*	1.219 (1.024∼1.450)*	1.269 (1.044∼1.544)*	1.255 (1.035∼1.522)*
25–36 month age	1.787 (1.340∼2.383)***	1.754 (1.322∼2.326)***	1.877 (1.376∼2.561)***	1.855 (1.359∼2.532)***	1.295 (1.074∼1.561)**	1.249 (1.036∼1.505)*	1.331 (1.078∼1.643)**	1.286 (1.043∼1.586)*
>36 month age	1.986 (1.175∼3.356)**	1.892 (1.123∼3.188)*	2.072 (1.227∼3.496)**	2.000 (1.187∼3.372)**	1.291 (0.844∼1.975)	1.221 (0.797∼1.870)	1.338 (0.866∼2.066)	1.271 (0.821∼1.968)
**Gross motor impairment** (**Aiming and catching**)								
Month age	1.028 (1.019∼1.037)***	1.029 (1.021∼1.039)***	1.024 (1.014∼1.034)***	1.024 (1.014∼1.034)***	1.015 (1.008∼1.023)***	1.015 (1.008∼1.023)***	1.014 (1.006∼1.021)***	1.013 (1.005∼1.020)**
≤12 month age	Ref	Ref	Ref	Ref	Ref	Ref	Ref	Ref
13–24 month age	1.487 (1.187∼1.861)***	1.580 (1.266∼1.971)***	1.303 (1.013∼1.677)*	1.315 (1.017∼1.699)*	1.437 (1.190∼1.736)***	1.473 (1.212∼1.790)***	1.375 (1.112∼1.700)**	1.368 (1.097∼1.706)**
25–36 month age	2.072 (1.558∼2.756)***	2.157 (1.630∼2.855)***	1.814 (1.339∼2.457)***	1.788 (1.320∼2.422)***	1.582 (1.276∼1.962)***	1.579 (1.271∼1.961)***	1.518 (1.217∼1.893)***	1.468 (1.175∼1.834)***
>36 month age	2.262 (1.439∼3.557)***	2.509 (1.579∼3.986)***	1.964 (1.222∼3.158)**	2.051 (1.260∼3.339)**	1.650 (1.125∼2.420)*	1.710 (1.163∼2.514)**	1.592 (1.078∼2.352)*	1.601 (1.078∼2.377)*
**Balance impairment**								
Month age	1.024 (1.013∼1.034)***	1.021 (1.010∼1.031)***	1.020 (1.009∼1.031)***	1.016 (1.005∼1.027)**	1.010 (1.003∼1.017)**	1.009 (1.002∼1.016)*	1.009 (1.001∼1.017)*	1.008 (1.000∼1.016)*
≤12 month age	Ref	Ref	Ref	Ref	Ref	Ref	Ref	Ref
13–24 month age	1.318 (0.963∼1.806)	1.288 (0.947∼1.752)	1.166 (0.844∼1.611)	1.120 (0.816∼1.536)	1.224 (1.037∼1.446)*	1.216 (1.031∼1.433)*	1.212 (0.990∼1.483)	1.211 (0.992∼1.478)
25–36 month age	1.855 (1.359∼2.533)***	1.720 (1.263∼2.343)***	1.610 (1.159∼2.235)**	1.467 (1.061∼2.028)*	1.272 (1.025∼1.579)*	1.251 (1.002∼1.562)*	1.248 (0.970∼1.606)	1.237 (0.954∼1.604)
>36 month age	1.587 (0.841∼2.995)	1.451 (0.777∼2.710)	1.424 (0.739∼2.743)	1.276 (0.670∼2.429)	1.410 (0.965∼2.062)	1.349 (0.926∼1.965)	1.388 (0.942∼2.045)	1.341 (0.913∼1.970)

*^a^Not adjusted for other variables.*

*^b^Adjusted for child characteristics.*

*^c^Adjusted for family characteristics.*

*^d^Adjusted for child and family characteristic.*

*p < 0.05, **p < 0.01, ***p < 0.001.

**FIGURE 2:**
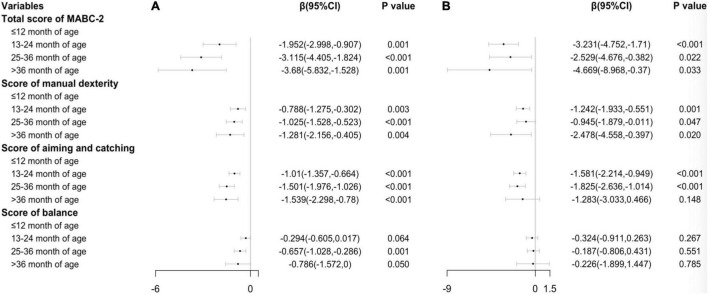
Associations between timing of independent eating and motor performance in **(A)** (one-child family, *n* = 9522) and **(B)** (two-child families, *n* = 2205) Ref: ≤ 12 month age.

**FIGURE 3:**
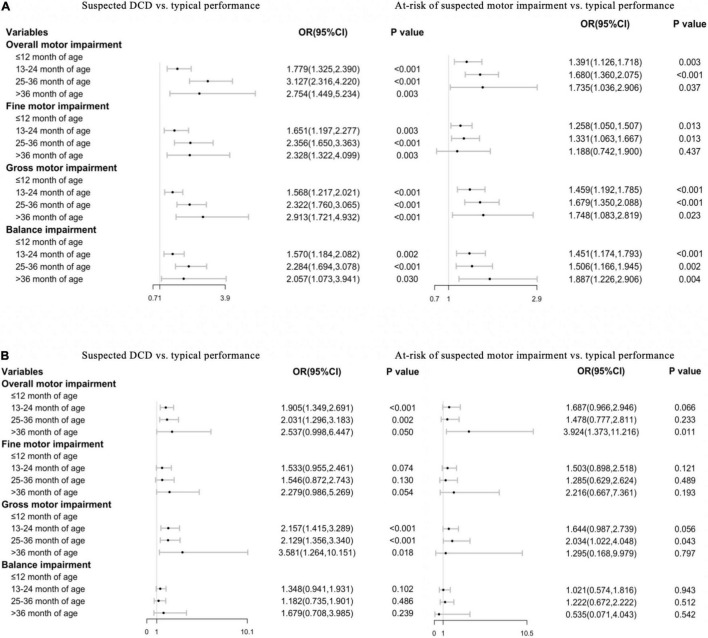
Associations between timing of independent eating and risk of DCD in **(A)** (one-child family, *n* = 9522) and **(B)** (two-child families, *n* = 2205) Ref: ≤ 12 month age.

Additionally, we analyzed the association between the onset of self-feeding and motor performance and impairment by stratifying the dataset by one-child and two-child families. Figure 2 showed that most results of the associations between the onset of self-feeding and MABC-2 scores remain statistically significant in one-child families, but were not statistically significant (each *p* > 0.05) in scores of balance (all age bands). Figure 3 showed that the associations between the onset of self-feeding and impairments of fine motor and balance disappeared in the two-child families (each *p* > 0.05 in all age bands).

## Discussion

The current prospective study used the largest population sample to date assessed by the diagnostic test (MABC-2) to measure motor impairment, providing a reliable findings that demonstrate a longitudinal link between the onset of self-feeding in early childhood and subsequent motor performance. Children with a delayed onset of self-feeding have an increased risk of suspected DCD. However, the likelihood that the delayed onset of self-feeding predicts poor motor performance was mitigated, specifically in the fine motor and balance performance subtasks, by the presence of a sibling.

Our study found an association between self-feeding onset and later motor performance in gross and fine motor, and balance performance as measured by MABC-2. The association can be explained by two possible reasons. First, self-feeding skills such as using cutlery occur in a set environment and may be more easily automated if they are practised daily from an early age. Self-feeding is one of the major motor activities in a child’s early life, and children with delayed onset of self-feeding have less life experience and practice in independent eating activities, which can, in turn, lead to poorer motor performance in later childhood. Secondly, environmental influences such as parents may also exert a powerful impact on the relationship between the onset of self-feeding and later motor performance. Parents can play an important role in encouraging children and providing training to children when they start to learn to eat independently. The same parents who tend to provide more support and positive feedback in children’s early self-feeding activities may also be the ones who can make a more positive impact on children’s motor development, including providing more training, resources, positive feedback, and other social interactions which can help children to achieve better motor performance (30). In this study, socioeconomic status (SES) was controlled by mediating parents’ education and income level, however, direct evidence is required in a future study on the association between parental circumstances and motor development in early childhood.

The most novel finding of our study is that the delayed onset of self-feeding can predict subsequent motor impairment. It has been reported that children with DCD showed difficulties in eating with a knife and fork, and displayed messy eating behaviors (31). A key component required in self-feeding is the ability to use cutlery effectively (12, 32), which requires a range of upper body and fine motor movements (13). It has been reported that DCD children were more likely to have the difficulty in maintaining their posture in sitting, and are inclined to rock or lean forward on their chair, constantly changing position while eating (6). Children with DCD can have neurological soft signs (e.g., hypotonia, persistence of primitive reflexes and immature balance) from a young age, which might interfere with their motor development (33). This can lead to difficulty following motor commands, planning and executing motor activities, and impaired performance in gross, fine motor and balance (6, 33, 34). Therefore, children with delayed onset of self-feeding may be impaired in a range of motor skills that are necessary for self-feeding, which might be observed from infancy, and children at risk of motor impairments can be identified at a very early stage in their lives (3).

The results of our study also showed that the presence of a sibling can reduce the strength of the association between the onset of self-feeding and motor impairment.

It has been reported that many parents and children can develop coping strategies to lessen the impact of motor difficulties on participation in activities of daily living. Previous research also reported a higher prevalence in the children from a one-child family compared to the children with a sibling (35), suggesting that the presence of a sibling is a protective factor to DCD. Children with a sibling are more likely to imitate the actions of other children than those without a sibling (36). Siblings are more likely to engage in play that provides motor experiences to children with motor impairment, which helps them to build motor proficiency (37). Additionally, older siblings can facilitate the onset of their younger siblings’ motor milestones (38), and play a key role in sports expertise development (39). Previous research has also shown significant sibling resemblance in gross motor coordination performance (40). Therefore, the presence of a sibling can either facilitate the onset of self-feeding, and/or positively influence the development of motor skills. It should be noticed that only fine motor and balance skills, but not aiming and catching skills, as measured with MABC-2 were mediated by the presence of a sibling. The results suggest that the presence of a sibling can change the motor developmental pattern, and may have different levels of influence in different motor domains. When the child has a sibling, the family environment can become more complex, and the influence of the child’s siblings, as well as that of the parents, must be considered. The role of a sibling, the characteristics of sibling – such as the birth order position of a child – the age difference between a child and the sibling, and the interaction pattern with the parents that is changed by the presence of a sibling should all be further examined in future research in motor development.

In conclusion, children who had a delayed self-feeding onset are more likely to develop DCD. There are two key implications from this study that relate to practice. First, a delay in the onset of self-feeding could be a marker for subsequent DCD/motor impairment, and the onset of self-feeding can be used as an important developmental milestone to facilitate the early identification of children with a motor impairment. Second, the presence of a sibling can reduce the strength of the association between the onset of self-feeding and the risk of motor impairment, and interventions targeted at supporting self-feeding and motor coordination of children from one-child families could help mitigate the risk of their motor impairment.

## Strengths and Limitations

In the current study, we used the onset time of self-feeding reported by parents as the indicator of children’s early motor developmental milestone. However, it could be argued that the reports (onset time of self-feeding) by parents might produce a recall bias. However, the bias can be mitigated when we use a prospective data-linkage study. The study involved a relatively large sample, and a wide range of relevant variables including child and family characteristics were measured and controlled for in the analysis. A large prospective sample can not only prevent the possible errors in parents estimated reports regarding the children’s motor development history, but can also limit the impact of different daily motor experiences and training in childhood. A large sample allows a simplified indicator to be used (i.e., the onset time of self-feeding without other descriptive information regarding the early eating behaviors), and provides guidance to facilitate the early identification of children with motor impairments (i.e., when is the expected age range of a child starting to self-feed). Additionally, we conducted the study in urban cities in southeast China (Jiangsu Province). Future studies could explore the associations between early eating behavior and childhood motor impairment in populations with varied socioeconomic statuses and culture backgrounds.

## Data Availability Statement

The original contributions presented in the study are included in the article/Supplementary Material, further inquiries can be directed to the corresponding authors.

## Ethics Statement

The study involving human participants was approved by the Local Education Board and Ethics Committee of the Children’s Hospital of Suzhou University. Parental consent and children’s assent was obtained before the investigation and tests. All information acquired was kept confidential and was accessible only to the researchers. Written informed consent to participate in this study was provided by the participants’ legal guardian/next of kin.

## Author Contributions

JH conceptualized and designed the study, drafted the initial manuscript, and reviewed and revised the manuscript. GW and AB were responsible for the critical revision of the manuscript for important intellectual content. JC, MX, YZ, and JZ completed the acquisition, analysis, or interpretation of data. HJ and GG designed the data collection instruments, collected data, carried out the initial analyses, and reviewed and revised the manuscript. WD designed the study, supervised the data collection, revised the manuscript, and supervised the interpretation of the findings of the work. All authors contributed to the article and approved the submitted version.

## Conflict of Interest

The authors declare that the research was conducted in the absence of any commercial or financial relationships that could be construed as a potential conflict of interest.

## Publisher’s Note

All claims expressed in this article are solely those of the authors and do not necessarily represent those of their affiliated organizations, or those of the publisher, the editors and the reviewers. Any product that may be evaluated in this article, or claim that may be made by its manufacturer, is not guaranteed or endorsed by the publisher.

## Footnotes

1 According to the national policy by then, couples were allowed to have no more than 2 children, and only in certain circumstances, the third child was allowed. To avoid any potential influence of those circumstances, the children from families with more than 2 children were excluded from the current study.
