# Supplementary material for: Association of the Onset of Self-Feeding With Subsequent Suspected Developmental Coordination Disorder: A Prospective Cohort Study in China
Source: Front Psychiatry. 2022 May 6;13:818771. doi: 10.3389/fpsyt.2022.818771 (PMC9120420; doi:10.3389/fpsyt.2022.818771)
Supplement: Supplementary file 1 [file Table_1.DOCX]

**APPENDIX TABLE 1 | The rates of subtypes of motor impairment by children’s and family’s characteristics (n=11727)^a^**

| Characteristic | Manual dexterity | | |  | | Aiming and catching | | | |  | |  | | Balance | | |  | | |
| --- | --- | --- | --- | --- | --- | --- | --- | --- | --- | --- | --- | --- | --- | --- | --- | --- | --- | --- | --- |
|  | Suspected DCD  (n%) | At-risk of suspected DCD  (n%) | Typical performance  (n%) |  | Suspected DCD  (n%) | | At-risk of suspected DCD  (n%) | Typical performance  (n%) |  | | Suspected DCD  (n%) | | At-risk of suspected DCD  (n%) | | Typical performance  (n%) | | | |  |
| Children’s characteristic |  |  |  |  |  | |  |  |  | |  | |  | | |  | |  |  |
| Children’s age |  |  |  |  |  | |  |  |  | |  | |  | | |  | |  |  |
| 3 | 29(5.3)*** | 103(9.1) | 932(9.3) |  | 152(19.1) | | 195(17.6) | 717(7.3) |  | | 83(15.9)*** | | 107(9.4) | | | 874(8.8) | |  |  |
| 4 | 184(33.5) | 432(38.4) | 2850(28.3) |  | 379(47.8) | | 431(38.9) | 2657(27.0) |  | | 225(43.2) | | 302(26.4) | | | 2940(29.2) | |  |  |
| 5 | 185(33.8) | 323(28.7) | 3833(38.1) |  | 143(18.0) | | 234(21.1) | 3964(40.30 |  | | 121(23.2) | | 270(23.6) | | | 3949(39.2) | |  |  |
| 6 | 150(27.4) | 268(23.8) | 2438(24.3) |  | 120(15.1) | | 248(22.4) | 2488(25.4) |  | | 92(17.7) | | 465(40.6) | | | 2299(22.8) | |  |  |
|  |  |  |  |  |  | |  |  |  | |  | |  | | |  | |  |  |
| Gender |  |  |  |  |  | |  |  |  | |  | |  | | |  | |  |  |
| Male | 370(67.5)*** | 670(59.4) | 5419(53.9) |  | 401(50.6) | | 597(53.9) | 5461(55.6) |  | | 351(67.4)*** | | 726(63.5) | | | 5382(53.5) | |  |  |
| Female | 178(32.5) | 457(40.6) | 4633(46.1) |  | 392(49.4) | | 511(46.1) | 4365(44.4) |  | | 170(32.6) | | 417(36.5) | | | 4681(46.5) | |  |  |
|  |  |  |  |  |  | |  |  |  | |  | |  | | |  | |  |  |
| Present BMI |  |  |  |  |  | |  |  |  | |  | |  | | |  | |  |  |
| ≤18 | 501(91.6) | 1048(93.0) | 9098(90.5) |  | 744(93.7)*** | | 1040(94.0) | 8863(90.2) |  | | 468(92.3) | | 1041(89.9) | | | 9138(90.8) | |  |  |
| >18 | 46(8.4) | 79(7.0) | 955(9.5) |  | 50(6.3) | | 66(6.0) | 964(9.8) |  | | 39(7.7) | | 117(10.1) | | | 925(9.2) | |  |  |
|  |  |  |  |  |  | |  |  |  | |  | |  | | |  | |  |  |
| Right handedness |  |  |  |  |  | |  |  |  | |  | |  | | |  | |  |  |
| No | 522(95.3) | 1078(95.7) | 9622(95.7) |  | 760(95.8) | | 1061(95.8) | 9402(95.7) |  | | 504(96.7) | | 1093(95.6) | | | 9626(95.7) | |  |  |
| Yes | 26(4.7) | 48(4.3) | 431(4.3) |  | 33(4.2) | | 47(4.2) | 424(4.3) |  | | 17(3.3) | | 50(4.4) | | | 437(4.3) | |  |  |
|  |  |  |  |  |  | |  |  |  | |  | |  | | |  | |  |  |
| Eyesight |  |  |  |  |  | |  |  |  | |  | |  | | |  | |  |  |
| Normal | 477(87.0)*** | 1009(89.6) | 8994(89.5) |  | 703(88.7)*** | | 996(89.9) | 8781(89.4) |  | | 449(86.2)*** | | 1008(88.2) | | | 9022(89.7) | |  |  |
| Abnormal | 71(13.0) | 117(10.4) | 1059(10.5) |  | 90(11.3) | | 112(10.1) | 1045(10.6) |  | | 72(13.8) | | 135(11.8) | | | 1041(10.3) | |  |  |
|  |  |  |  |  |  | |  |  |  | |  | |  | | |  | |  |  |
| Gestational weeks |  |  |  |  |  | |  |  |  | |  | |  | | |  | |  |  |
| <37 | 40(7.3) | 93(8.3) | 816(8.1) |  | 58(7.3) | | 107(9.7) | 784(8.0) |  | | 44(8.4) | | 90(7.9) | | | 815(8.1) | |  |  |
| ≥37 | 508(92.7) | 1034(91.7) | 9236(91.9) |  | 735(92.7) | | 1001(90.3) | 9042(92.0) |  | | 477(91.6) | | 1053(92.1) | | | 9248(91.9) | |  |  |
|  |  |  |  |  |  | |  |  |  | |  | |  | | |  | |  |  |
| Birth weight |  |  |  |  |  | |  |  |  | |  | |  | | |  | |  |  |
| <2500g | 15(2.7) | 25(2.2) | 466(4.6) |  | 22(2.8) | | 26(2.3) | 458(4.7) |  | | 14(2.7) | | 30(2.6) | | | 462(4.6) | |  |  |
| ≥2500g | 533(97.3) | 1102(97.8) | 9586(95.4) |  | 771(97.2) | | 1083(97.7) | 9368(95.3) |  | | 507(97.3) | | 1113(97.4) | | | 9601(95.4) | |  |  |
|  |  |  |  |  |  | |  |  |  | |  | |  | | |  | |  |  |
| Family’s characteristics |  |  |  |  |  | |  |  |  | |  | |  | | |  | |  |  |
| Higher education of mother |  |  |  |  |  | |  |  |  | |  | |  | | |  | |  |  |
| No | 319(58.2)** | 555(49.3) | 4578(45.5) |  | 411(51.8)* | | 512(46.2) | 4568(46.3) |  | | 242(46.4)*** | | 491(43.0) | | | 4719(46.9) | |  |  |
| Yes | 229(41.8) | 571(50.7) | 5475(55.5) |  | 382(48.2) | | 596(53.8) | 5298(53.7) |  | | 280(53.6) | | 652(57.0) | | | 5343(53.1) | |  |  |
|  |  |  |  |  |  | |  |  |  | |  | |  | | |  | |  |  |
| Higher education of father |  |  |  |  |  | |  |  |  | |  | |  | | |  | |  |  |
| No | 288(52.6)*** | 473(42.0) | 3634(35.1) |  | 338(42.6) | | 440(39.7) | 3619(36.8) |  | | 204(39.2) | | 410(35.9) | | | 3682(37.0) | |  |  |
| Yes | 260(47.4) | 653(58.0) | 6718(64.9) |  | 455(57.4) | | 669(60.3) | 6207(63.2) |  | | 317(60.8) | | 733(64.1) | | | 6281(67.0) | |  |  |
|  |  |  |  |  |  | |  |  |  | |  | |  | | |  | |  |  |
| Family annual per-capita income (RMB) ^a^ |  |  |  |  |  | |  |  |  | |  | |  | | |  | |  |  |
| Below | 410(74.8)* | 838(74.4) | 7325(72.9) |  | 623(78.6)*** | | 855(77.2) | 7096(72.2) |  | | 427(82.0) | | 840(73.5) | | | 7306(72.6) | |  |  |
| Above or equal to | 138(25.2) | 288(25.6) | 2727(27.1) |  | 170(21.4) | | 253(22.8) | 2730(27.8) |  | | 94(18.0) | | 303(26.5) | | | 2756(27.4) | |  |  |
|  |  |  |  |  |  | |  |  |  | |  | |  | | |  | |  |  |
| Family structure |  |  |  |  |  | |  |  |  | |  | |  | | |  | |  |  |
| Single families | 14(2.6) | 17(1.5) | 126(1.3) |  | 9(1.2) | | 16(1.4) | 132(1.3) |  | | 8(1.5)* | | 23(2.0) | | | 127(1.3) | |  |  |
| Nuclear families | 376(68.6) | 737(65.5) | 6395(63.6) |  | 519(65.4) | | 728(65.7) | 6260(63.7) |  | | 315(60.3) | | 728(63.7) | | | 6465(64.2) | |  |  |
| Extended families | 158(28.8) | 372(33.0) | 3532(35.1) |  | 265(33.4) | | 364(32.9) | 3434(34.9) |  | | 199(38.2) | | 392(34.3) | | | 3471(34.5) | |  |  |
|  |  |  |  |  |  | |  |  |  | |  | |  | | |  | |  |  |
| The number of children in the family ^a^ |  |  |  |  |  | |  |  |  | |  | |  | | |  | |  |  |
| One | 418(76.30 | 888(78.9) | 8144(81.1) |  | 611(77.0) | | 872(78.7) | 7967(81.1) |  | | 432(82.9)*** | | 923(80.8) | | | 8095(80.4) | |  |  |
| Two | 130(23.7) | 238(21.1) | 1903(18.9) |  | 182(23.0) | | 236(21.3) | 1859(18.9) |  | | 89(17.1) | | 220(19.2) | | | 1968(19.6) | |  |  |
|  |  |  |  |  |  | |  |  |  | |  | |  | | |  | |  |  |
| Maternal age at birth |  |  |  |  |  | |  |  |  | |  | |  | | |  | |  |  |
| <30 | 458(83.6) | 989(87.8) | 8599(85.5) |  | 667(84.1) | | 955(86.2) | 8425(85.7) |  | | 448(86.0) | | 956(83.6) | | | 8642(85.9) | |  |  |
| 30-34 | 65(11.9) | 101(9.0) | 1170(11.6) |  | 94(11.9) | | 109(9.8) | 1133(11.5) |  | | 50(9.6) | | 145(12.7) | | | 1141(11.3) | |  |  |
| ≥35 | 25(4.5) | 36(3.2) | 284(2.8) |  | 32(4.0) | | 44(4.0) | 268(2.7) |  | | 23(4.4) | | 42(3.7) | | | 280(2.8) | |  |  |
|  |  |  |  |  |  | |  |  |  | |  | |  | | |  | |  |  |
| Maternal complications during pregnancy |  |  |  |  |  | |  |  |  | |  | |  | | |  | |  |  |
| No | 458(83.6) | 917(81.4) | 8253(82.1) |  | 619(78.1) | | 897(80.9) | 8112(82.6) |  | | 413(79.3) | | 916(80.1) | | |  | |  |  |
| Yes | 90(16.4) | 209(18.6) | 1799(17.9) |  | 174(21.9) | | 212(19.1) | 1714(17.4) |  | | 108(20.7) | | 227(19.9) | | | 8299(82.5) | |  |  |

^a^ Pearson chi-square test

^b^ The national average family per-capita income in the last year of the survey time

^c^ Having one of maternal complications during pregnancy including intrauterine distress, asphyxia, cerebral hemorrhage, encephalitis, convulsions and lung diseases

**p*<0.05, ***p*<0.01,****p<*0.001
